# Methodologies for Monitoring Mental Health on Twitter: Systematic Review

**DOI:** 10.2196/42734

**Published:** 2023-05-08

**Authors:** Nina H Di Cara, Valerio Maggio, Oliver S P Davis, Claire M A Haworth

**Affiliations:** 1 School of Psychological Science University of Bristol Bristol United Kingdom; 2 MRC Integrative Epidemiology Unit University of Bristol Bristol United Kingdom; 3 Population Health Sciences Bristol Medical School University of Bristol Bristol United Kingdom; 4 The Alan Turing Institute London United Kingdom

**Keywords:** social media, mental health, mental illness, machine learning

## Abstract

**Background:**

The use of social media data to predict mental health outcomes has the potential to allow for the continuous monitoring of mental health and well-being and provide timely information that can supplement traditional clinical assessments. However, it is crucial that the methodologies used to create models for this purpose are of high quality from both a mental health and machine learning perspective. Twitter has been a popular choice of social media because of the accessibility of its data, but access to big data sets is not a guarantee of robust results.

**Objective:**

This study aims to review the current methodologies used in the literature for predicting mental health outcomes from Twitter data, with a focus on the quality of the underlying mental health data and the machine learning methods used.

**Methods:**

A systematic search was performed across 6 databases, using keywords related to mental health disorders, algorithms, and social media. In total, 2759 records were screened, of which 164 (5.94%) papers were analyzed. Information about methodologies for data acquisition, preprocessing, model creation, and validation was collected, as well as information about replicability and ethical considerations.

**Results:**

The 164 studies reviewed used 119 primary data sets. There were an additional 8 data sets identified that were not described in enough detail to include, and 6.1% (10/164) of the papers did not describe their data sets at all. Of these 119 data sets, only 16 (13.4%) had access to ground truth data (ie, known characteristics) about the mental health disorders of social media users. The other 86.6% (103/119) of data sets collected data by searching keywords or phrases, which may not be representative of patterns of Twitter use for those with mental health disorders. The annotation of mental health disorders for classification labels was variable, and 57.1% (68/119) of the data sets had no ground truth or clinical input on this annotation. Despite being a common mental health disorder, anxiety received little attention.

**Conclusions:**

The sharing of high-quality ground truth data sets is crucial for the development of trustworthy algorithms that have clinical and research utility. Further collaboration across disciplines and contexts is encouraged to better understand what types of predictions will be useful in supporting the management and identification of mental health disorders. A series of recommendations for researchers in this field and for the wider research community are made, with the aim of enhancing the quality and utility of future outputs.

## Introduction

### Background

The detection of signals of mental health through big data is a rapidly evolving field of research that requires interdisciplinary expertise, from the behavioral psychology of mental health, to communication science, to the computational modeling of associated behaviors using data [1]. Social media has been a popular platform for accessing data to investigate these digital signals [2,3] and has provided a promising opportunity to model individual and interpersonal behaviors to further understand typically private topics such as hate speech [4] and political ideation [5] as well as mental health. Although there is a range of possible social media platforms that could be used for analysis, Twitter has been a popular choice for research because of its public-facing design and readily available application programming interface (API), which, until recent changes to the Twitter API, have enabled easy access to data for research [6,7].

Currently, mental illness is one of the leading causes of the overall global disease burden [8], with depression estimated to be one of the most prevalent diseases worldwide [9]. The implications of mental ill health are profound on both a micro and macro scale, from personal relationships to the global economy [10,11]. As a result, there has been increasing interest in the potential of data-driven methods to provide a new approach to the early detection and prevention of mental health disorders [12-16], particularly for young people [13], which could serve to promote access to mental health care and improve opportunities for clinical or self-monitoring. The use of data created through day-to-day technology use could even contribute to clinical assessments by health care professionals, who typically use questionnaire-style diagnostic tools that can be biased by a patient’s retrospective recall [17] and so cannot always provide an accurate overview of a patient’s well-being for weeks, or months, at a time. Additional benefits of using social media data are the ability to collect data on populations with less common mental health disorders such as schizophrenia or posttraumatic stress disorder (PTSD), which is generally not possible outside a clinical environment.

### Themes From Previous Reviews

There has been a series of reviews on the topic of mental health inference from social media, all of which have focused on a range of social media platforms. The key reviews identified were by Wongkoblap et al [7] in 2017, Guntuku et al [18] in 2017, Chancellor and De Choudhury [19] in 2020, and Kim et al [20] in 2021. Despite the potential for digital footprint data to drive advances in the monitoring and detection of mental health outcomes, previous research and reviews in the field have raised substantial concerns about the current literature. These concerns center on the validity of ground truth mental health data, methodological clarity, and the ethics of the research and its proposed applications.

First, there have been concerns about the quality of the data used to train models for mental health inference owing to poor construct validity in the generation of data labels [7,19,21]. For machine learning to be effective, the labels that a supervised learning algorithm should be “learning” from (ie, the ground truth) should represent the same construct that the researcher intends for the model to predict in the future; construct validity refers to this equivalence between the label and the construct to be predicted. Systematic reviews by both Wongkoblap et al [7] and Chancellor and De Choudhury [19] found that using mentions of mental health disorders and affiliations was a very common method for constructing data sets. This means that studies use data sets for training that are constructed and labeled based on mentions of mental health disorders in tweets (eg, a user tweeting “I have depression”) or based on affiliations with accounts about a specific disorder (such as following an account that tweets about experiences of PTSD) [7,19], which both make assumptions about users rather than having externally validated information about whether the user does actually have a mental health disorder. Research by Ernala et al [21] showed that, although positive cases identified through affiliations and mentions of disorders led to fairly good performance for schizophrenia prediction when validated on the same data set, they performed poorly when validated against a separate data set where diagnoses had been assigned by clinicians. The poor performance of models using assumed ground truth information when tested on clinically validated ground truth suggests that the construct validity of using mentions of disorders and affiliations as the ground truth is likely to be unsatisfactory for transferring models to a real-world setting, although model overfitting is another potential issue to consider. Chancellor and De Choudhury [19] found that only 17 of the 75 studies they included used methods to obtain ground truth that had validity external to the training data set, such as from participants themselves, news reports of their deaths, or their medical records.

In addition to concerns regarding the data being used to train models in the literature, previous reviews [20] have also identified a lack of transparency and clarity in the methodologies used to produce models. It is common for researchers not to declare important details such as the features included in their models [19], and it is also uncommon for researchers to include data availability statements [7,22]. The review by Chancellor and De Choudhury [19] found that only 42% of the 75 papers included reported on all 5 of what they considered to be minimum reporting criteria, which were the number of samples or data points, the number of variables or features, the algorithm or regression chosen, at least one validation method, and their explicit fit or performance metrics. Overall, the lack of clarity and transparency makes it difficult to assess how research has been conducted and, therefore, compare results between papers and determine the quality of research methods [20].

Aligned with concerns about the sourcing of ground truth data, another issue that has been raised is the characterization of mental health in general, recognizing that the mathematical modeling of a psychological construct requires making assumptions about the way it can be captured as data [23]. For instance, representing mental health outcomes as binary implies certain assumptions about the way the researcher has chosen to model mental health as a construct, which does not allow for a range of symptom severity or for the possibility of comorbidity, which is generally high among common mental health disorders such as anxiety and depression [24,25]. This is then reflected in whether the task is posed as a classification or regression problem.

There are also assumptions regarding the decision on the nature of the target of an analysis. Chancellor et al [24] conducted a discourse analysis of the ways in which researchers wrote about the people behind the data being used in mental health inference from social media and found that it was often unclear whether the research considered people or individual tweets as unwell. Papers classifying individual tweets sometimes stated their results as classifying the mental health of a user when in fact they were classifying individual tweets. Notwithstanding that there is a considerable assumption in using a single tweet as an indication of depression, this also makes it challenging to understand both the analysis and the results of the proposed models, as what is being predicted—tweet or individual outcome—is unclear or not reported at all.

Finally, all previous reviews have highlighted ethics as an ongoing concern. The ethical concerns generally refer to the privacy of the individuals whose data are often being used without their knowledge or consent, the sharing of data sets that contain inferred information about those individuals (eg, a suspected mental health disorder), and the implications of sharing models that could publicly infer information about individuals who had no association with the original study. Outside the research itself, there are outstanding questions regarding the ethics of using the proposed systems in practice, such as the impact of misclassification on patients [18]. It is worth noting that these ethical concerns are also an ongoing discussion in the critical algorithm literature [26,27].

### The Purpose of This Study

The most recent systematic review that covered all papers published on the topic of predicting mental health from social media sites was the review by Chancellor and De Choudhury [19] in 2020. They proposed a list of modeling decisions and outcomes that should be reported in all studies to improve methodological clarity in response to their findings of insufficient method reporting across 57% of the 75 included studies. This review included literature up to 2018 and considered research on a range of 12 social media sites.

Since this review took place, there have been 4 years of new literature to account for. In this time, there has been a substantial trend in the sciences, especially psychology, toward open science and the improved sharing of data and methodological decisions fueled by the so-called reproducibility crisis [28,29]. Ethical concerns have also received greater attention in the past few years, especially in fields using social media data, in the wake of the Cambridge Analytica scandal. The scandal, which broke in 2018, revealed that millions of people’s Facebook data were used to analyze and infer their personal characteristics for political advertising without their consent. Given these wider cultural changes, the time since previous reviews, and also the opportunity for recommendations from previous reviews in 2017 [7,18] to have been incorporated into new research, we intended to provide an updated review in the area of mental health inference from social media. Specifically, this review focused on the social networking site Twitter as it includes the period in which research access to the Facebook and Instagram APIs, 2 of the most popular social media sites, was removed to provide tighter controls on user data. No such controls were implemented on Twitter.

In this review, we set out to understand the current scope, direction, and trends in the prediction of mental health outcomes from Twitter data. We conducted a review of the existing literature on the prediction of mental health disorders and mental well-being from Twitter by implementing a systematic search to find papers published between January 2013 and December 2021. Our aims were similar to those posed in previous reviews [7,18,19] in that they focused on methodological processes rather than the results of the research. We set out to evaluate (1) the machine learning methodologies used, such as the ways in which preprocessing, feature selection, modeling, and validation were conducted; (2) the data sets that were used in each study, such as how the data sets were collected and how mental health outcomes were labeled in these data sets to achieve construct validity; (3) the replicability of each study; and (4) whether each paper discussed any ethical considerations.

Uniquely, this review aimed to include well-being constructs as well as mental health disorders and also aimed to understand methods to construct data sets as separate from the methods to model mental health, which allowed for the analysis of the prevalence of data set reuse and popularity.

As is crucial in interdisciplinary work, we first wish to establish a shared understanding with the reader of the use of terminology throughout this paper [30]. Here, we take “prediction” to be an algorithmic decision to assign an unseen piece of data to a category (eg, depressed or not depressed) without meaning prediction of the future [31]. We also make distinctions between *mental health* and a *mental health disorder*, with the term *mental health disorder* reserved for references to a medical condition and being separate from but related to general mental health and well-being [32,33]. *Mental health outcomes* refers to both mental health disorders and specific well-being constructs (eg, general well-being, happiness, life satisfaction, or self-esteem).

## Methods

### Search Methodology

On May 7, 2019, and with 2 updated searches on October 26, 2019, and December 6, 2021, we conducted a search of 6 electronic databases (Web of Science; Scopus; PubMed; and Ovid MEDLINE, PsycINFO, and PsycArticles), as well as a Google Scholar Search. The search was for peer-reviewed articles or papers that contained terms related to mental health disorders and well-being, machine learning, and Twitter in their titles or abstracts (see Multimedia Appendix 1 for the full list of search terms). The search terms for machine learning and mental health were developed by initially putting together a list of mental health disorders and synonyms for “mental health disorders,” such as “mental illness,” or references to algorithmic methods; reviewing previous systematic reviews in this area for missed keywords; and presenting the results to colleagues who work in mental health and machine learning for feedback on the included terms. Each search was refined for the requirements of the database. The results were required to have been published in 2006 or later to avoid unrelated publications from before Twitter was created.

Several key review papers in the field of mental health prediction from social media were identified before the systematic review [7,18,19,34], and 16 other review papers in related fields were identified through the systematic review process [20,35-49]. Secondary citations from all these reviews were included in the screening phase if they had not already been identified through the database search. Relevant articles from the Workshop on Computational Linguistics and Clinical Psychology (CLPsych) conference proceedings (2014-2021) were also included if they had not already been identified. Finally, a small number of papers were identified through recommendations from colleagues and the referencing software Mendeley (Elsevier), which were added to the database search results to be screened for duplicates and relevance.

### Screening Methodology

The Rayyan software (Rayyan Systems, Inc) [50] was used to identify and remove duplicates from the results and review the titles and abstracts to screen papers for a full-text review. At this stage, papers that appeared to be irrelevant, for instance, related to personal social networks as opposed to web-based social networks or having no relevance to mental health, were removed. We also removed conference abstracts and theses but did include papers from conference proceedings and workshop tasks as these are a common format for developments in the computer sciences.

A full-text review was then conducted of the remaining 650 papers. At this stage, the inclusion criteria were as follows: (1) the study considered data from Twitter to build the algorithm (despite being similar to Twitter, Weibo was excluded because of some differences in the data types available and the nature of use); (2) the study did not consider a specific group of people, such as veterans or new mothers; (3) the study considered a mental health disorder or specific well-being construct rather than a less specific concept such as stress (this was based on the paper’s title and what it stated it predicted); and (4) the study trained a model for the purposes of inference rather than solely analysis of features.

This full-text review left 164 papers that met the criteria for inclusion in the analysis.

### Data Collection

The literature search, screening, and analysis were completed by ND. Details recorded for each study were the mental health outcome studied, machine learning algorithms used, features and model input, validation and evaluation strategies, and the reported results. For each primary data set identified, meaning those where data were collected by the research team and not reused from an existing study, we also recorded the method of data collection, the key characteristics of the data set, how the data were annotated, and any quality control processes used. A complete record of the identified and reviewed papers is included in the web-based Multimedia Appendix 2. The full data extraction details are provided in Multimedia Appendix 3.

## Results

### Overview

**Figure 1:**
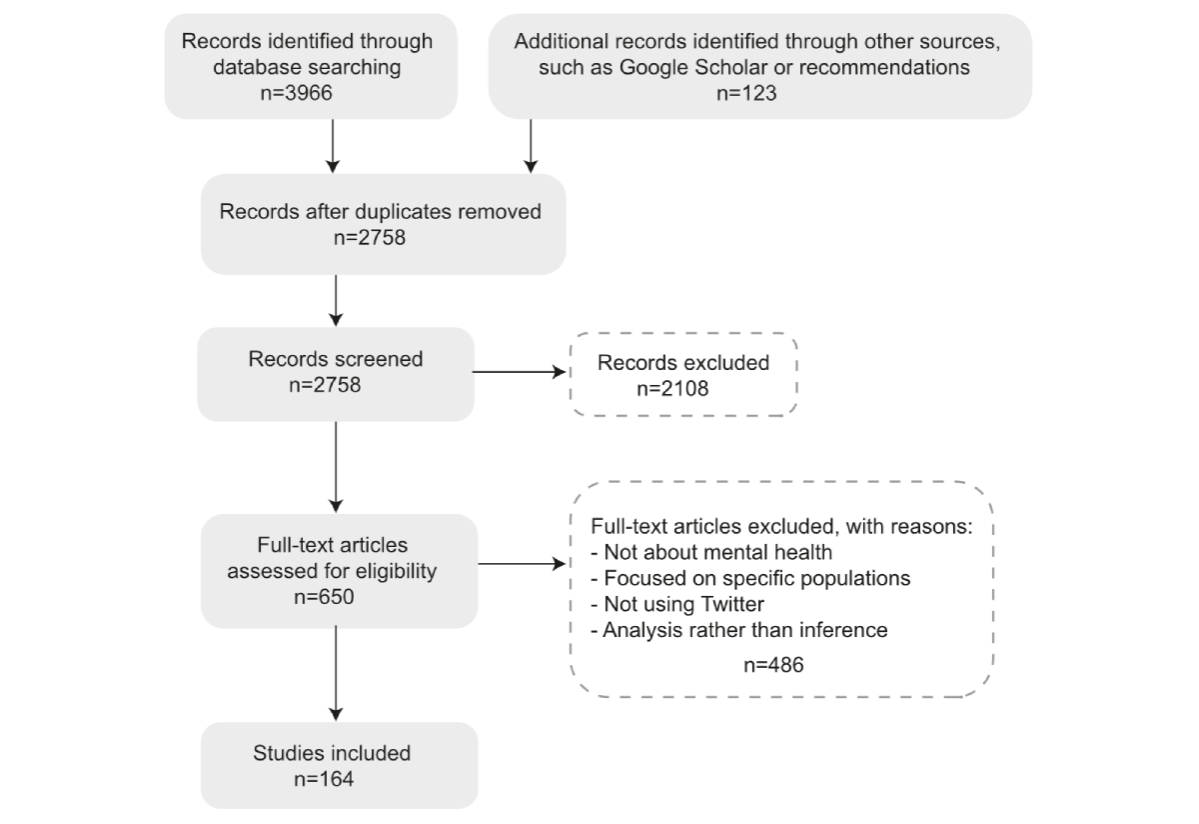
PRISMA (Preferred Reporting Items for Systematic Reviews and Meta-Analyses) flow diagram of inclusion and exclusion figures for the literature search.

Figure 1 illustrates the number of papers included at each stage of the screening process.

Table 1 shows how many of the papers included were published in each year and shows that 45.7% (75/164) of the papers identified on this topic were published from 2019 onward, which is after the range of dates included in previous reviews. Overall, of the 164 papers, 96 (58.5%) were from conference proceedings, 56 (34.1%) were journal articles, and 13 (7.9%) were from workshops.

**Table 1:** The number of papers included in the review that were published each year (N=164).

Year	Papers, n (%)
2013	3 (1.8)
2014	6 (3.7)
2015	11 (6.7)
2016	7 (4.3)
2017	16 (9.8)
2018	13 (7.9)
2019	33 (20.1)
2020	36 (22.0)
2021	39 (23.8)

### Mental Health Outcomes Predicted

**Figure 2:**
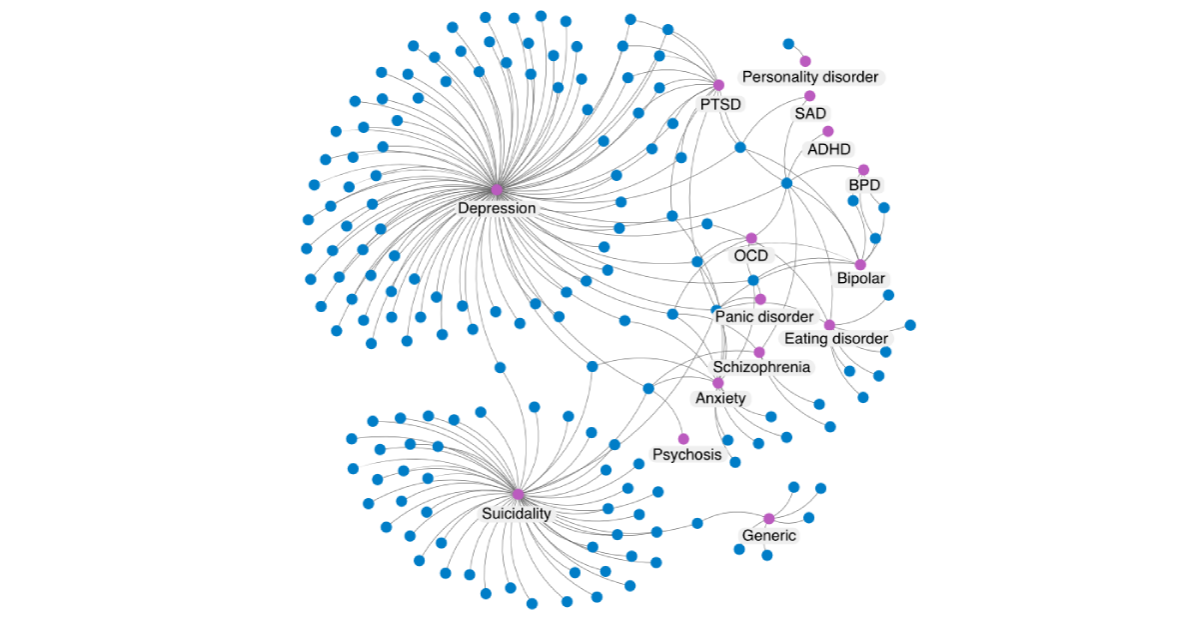
Network diagram showing which mental health disorder (pink) each study (blue) attempted to infer. Depression and suicidality were the most popular, with most studies attempting to predict a single outcome. ADHD: attention-deficit/hyperactivity disorder; BPD: borderline personality disorder; OCD: obsessive-compulsive disorder; PTSD: posttraumatic stress disorder; SAD: seasonal affective disorder.

Figure 2 outlines the network of mental health disorders that the included studies covered. It illustrates that depression was the most common target and was predicted in 56.7% (93/164) of the studies, followed by suicidality (50/164, 30.5%), PTSD (14/164, 8.5%), and anxiety (13/164, 7.9%). It was most common for studies to approach this problem as a single-class prediction, although 15.9% (26/164) of the studies considered more than one mental health disorder.

**Figure 3:**
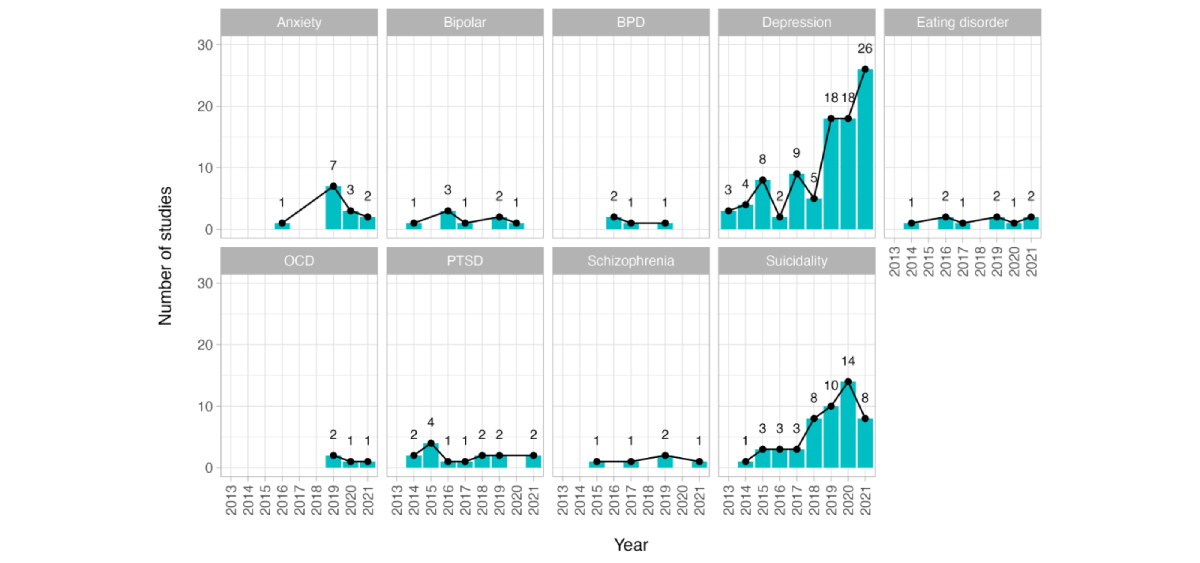
The number of studies considering each mental health disorder by year of publication (for disorders included in >2 studies). BPD: borderline personality disorder; OCD: obsessive-compulsive disorder; PTSD: posttraumatic stress disorder.

Figure 3 shows that there has been an increase since 2019 in the number of studies being published on this topic, but they are dominated by studies on depression and, to some extent, suicidality. The analysis of other disorders has remained fairly static or declined over time. Although there is an overall tendency to focus on mental health disorders, there was a study that included the prediction of happiness and self-esteem [51].

### Data Sets

#### Overview

One of the aims of this review was to analyze the unique data sets that were used for prediction of mental health outcomes across the included studies. Overall, we identified 127 unique data sets from 164 papers included in this review, which we will refer to as *primary* data sets; 6.1% (10/164) of the papers did not provide a description of the data set. Of these 127 unique data sets, 8 (6.3%) were not described in enough detail for analysis. This was usually due to links to data set sources being invalid or links to web-based data sets that were not actually described in the text. This left 119 unique data sets that contained enough details to be analyzed.

All the studies identified in this review (164/164, 100%) used an annotated data set to train the prediction models. *Annotation* refers to the process by which each observation or data point that will be used to train the model is given an outcome that the model is trained to predict. In this case, the annotations were expected to be a mental health outcome.

Different studies took different approaches to the process of collecting and annotating their data sets, and in this section, we provide an overview of these processes for the 119 data sets that were adequately described. Then, as some studies used primary data sets that were developed and shared by others, we also provide a brief description of the data sets that were most commonly reused.

#### Descriptions of Data Collection

To understand the approaches to data collection, we recorded whether the description of the data set specified the number of tweets included in the final data set, how many individual users were in the data set, the period over which Twitter data were collected, the API or tool used to access the Twitter data, and the search query or strategy used to collect the data. These were chosen as they represent basic descriptive information that is important for interpreting the results of the studies and also represent reasons why some studies may find differing results. For example, using data from different periods, different APIs, and different search queries to access data would result in different samples, and these may then yield different predictions when addressing the same core question.

From the descriptions of the 119 data sets included, we found that 57.1% (68/119) of the data sets included the number of users in the data set, 79.8% (95/119) included how many tweets were in the data set, 55.5% (66/119) included the period over which the data were collected from Twitter, 69.7% (83/119) included which API or tool was used to access the Twitter data, and 90.8% (108/119) included the search strategy they used to query the API. The smallest described data set was that of Coello-Guilarte et al [52] with 200 annotated tweets, and the largest was that of Shen et al [53] with >300 million tweets from users they determined to be depressed and 10 billion control tweets.

#### Annotating Mental Health Outcomes

Next, we recorded information on how the data were annotated using mental health labels. This included the method used to attribute labels to the tweets or users and whether there was any secondary quality control conducted by human annotators if an automated method was used. In addition, we evaluated the range of methods that were used to develop control samples of tweets or users who did not display the mental health outcome that was being predicted.

We originally intended to also record whether annotations were being made at the tweet or user level, but unfortunately, it was not common for studies to specify which of these approaches they were taking, and so it was not possible to summarize the frequencies observed in the papers reviewed.

As Table 2 illustrates, the data sets were annotated in many different ways, but only 13.4% (16/119) of the data sets overall were validated using offline ground truth. That is to say that the label was not assumed from the data collected. Even within those studies that did use validated scales for the ground truth, they could define the threshold score for the presence of a disorder from the same scale differently. For instance, a Center for Epidemiological Studies Depression Scale score of >30 or >22 were both used as cutoff scores for the classification of depression in different studies. Owing to the variety of methods presented, comparisons between studies could be between data sets that had very different definitions of the same mental health outcome.

Of the studies using keyword- or self-disclosure–based annotation, many (54/80, 68%) attempted to increase the accuracy of by introducing human annotators to the process. However, 3% (2/80) of the studies reported that annotators found it difficult to decide on the category that tweets should be placed in, especially when they were seen without the context of other tweets from the same user [54,55]. To overcome this, some annotated data sets used more than one annotator to assess agreement between annotators or introduced a third annotator to provide a deciding opinion on conflicting assessments [56,57]. As might be expected, there was generally a relationship between the size of a data set and the level of quality control; highly curated data with labels produced by experts and multiple coders tended to be smaller in volume, and those using largely automated methods were able to produce vast data sets with little human input on the target classification labels.

Most studies (147/164, 89.6%) defined mental health as a binary or categorical outcome as opposed to using a continuous scale (9/164, 5.5%), and 4.9% (8/164) not specifying their methods in enough detail to be certain. This is important as the outcome being predicted indicates a different research question and, ultimately, a different purpose, for instance, classification of tweets that are “risky” or “not risky” in terms of suicidal expression versus a longitudinal view of change in depressive symptoms. This was largely influenced by the approaches to data labeling, where the presence of keywords or self-disclosure does not allow for a measurement of symptom intensity and instead necessitates a binary or categorical approach.

As most data sets (147/164, 89.6%) took a categorical approach to mental health, there were a variety of approaches to developing a control sample. These included taking a random sample of tweets from the Streaming API on a particular day, searching for a word or phrase (such as “the” or “today is my birthday”) in the Search API and using the results as controls, or simply using all the users who were not labeled as positive from the original keyword or phrase search. In some instances, studies conducted checks to ensure that there were no overlaps between the positive and negative samples, but this was not always stated as being the case. In terms of the balance between cases and controls in the data sets, 2 main approaches were to intentionally balance cases and controls [58-68], or to use the chosen criteria to find the “naturally occurring” number of cases from their data set [6,53,55,64,69-71].

**Table 2:** Overview of the different methods used to annotate data sets with ground truth labels.

Ground truth type			Description		Count		QC^a^		Examples
**Validated^b^**									
	Self-report	Completion of a standardized measure or disclosure of affected periods by the individual		12		N/A^c^		User scored >30 in the CES-D^d^ or CES-D score used as a continuous variable	
	Secondary report	News reports of death by suicide or data donation by family following death		4		N/A		Name reported in the media was searched on Twitter for a user account	
**Data-driven^e^**									
	Affiliation	The account either followed or interacted with a system or other accounts known to be associated with the mental health disorder being considered		2		N/A		Accounts that had retweeted tweets from a list of accounts about depression were annotated as being depressed	
	Keywords	A certain number or combination of keywords used to search the Twitter API^f^ believed to indicate the presence of the mental health disorder		51		Expert: 20; nonexpert: 18; none: 13^g^		User used the string “depress” >5 times in 2 weeks, and their timeline was reviewed by a clinical psychologist to confirm that the assessment was reasonable (expert QC), or the user used “depression” at least once in a tweet (no QC)	
	Self-disclosure	A phrase such as “I have been diagnosed with X” was used to search the Twitter API and used to indicate the presence of the mental health disorder		29		Expert: 2; nonexpert: 14; none: 13		String “I have been diagnosed with depression” was used without checking the context (no QC), or the string “I have been diagnosed with depression” was used following verification by a clinical psychologist (expert QC) or a computer science researcher (nonexpert QC)	
	Sentiment label	Some threshold was decided based on a sentiment polarity score that mapped it to a mental health outcome		2		N/A		Sentiment score of <−1 meant that the user was annotated as depressed	
**Other**									
	Random sample	A random sample of tweets was taken from the Streaming API or based on some other criteria, such as a particular language being used, and screened for inclusion		5		N/A		Tweets in a particular language were accessed from the Streaming API and annotated as suicidal if the researcher thought they indicated suicidality	
	Unknown	Not enough information provided to understand the method for generating ground truth labels		14		N/A		N/A	

^a^QC: quality control.

^b^“Validated” refers to data annotations that were not assumed from the data collected and were validated by either the user themself or an external source.

^c^N/A: not applicable.

^d^CES-D: Center for Epidemiological Studies Depression Scale.

^e^“Data-driven” refers to annotations that were derived from the data collected from social media.

^f^API: application programming interface.

^g^Expert annotation was performed by those who were called experts in the paper or who were reported as having some academic or practical background in mental health practice. Nonexpert annotation was performed by anyone not in the *Expert* category, for instance, undergraduate students or computer science researchers.

#### Data Set Reuse

Of the 119 primary data sets identified, there were 2 (1.7%) that were reused more often than others. The data set on depression and PTSD, which was produced for the CLPsych workshop in 2015 [64], was used a total of 10 times, and the data set produced by Shen et al [53] for depression prediction in 2017 was used the most often at 14 times. The other most frequently reused data sets were those produced by Burnap et al [72] in 2017 for suicidality (4 uses), by Jamil et al [73] in 2017 for depression (3 uses), and by Vioules et al [74] in 2018 for suicidality (3 uses). Another data set used in 2.4% (4/164) of the studies despite not being created for mental health prediction was the “sentiment140” data set. This is a Kaggle (a website where individuals and teams can participate on the web in data science challenges) competition data set where tweets are labeled with their sentiment polarity.

Finally, the remaining data sets were created by the authors for their own use and occasionally reused by the same authors over 2 studies. In most cases, data sets were created specifically for the task the study was focused on. These included data sets of tweets in other languages, such as Spanish [52], Bengali [75], Japanese [51], and Arabic [76], as well as English, which was the most common language studied.

### Modeling Workflows

#### Overview

After identifying the training data set, there are typically a series of stages to go through to develop and assess a predictive model. First, the researcher must prepare the data set for use (known as preprocessing); select the features that will be used in the model (known as feature selection); choose and apply an algorithm to create a model from; and then, finally, validate the model to assess how well it performs on unseen data.

**Figure 4:**
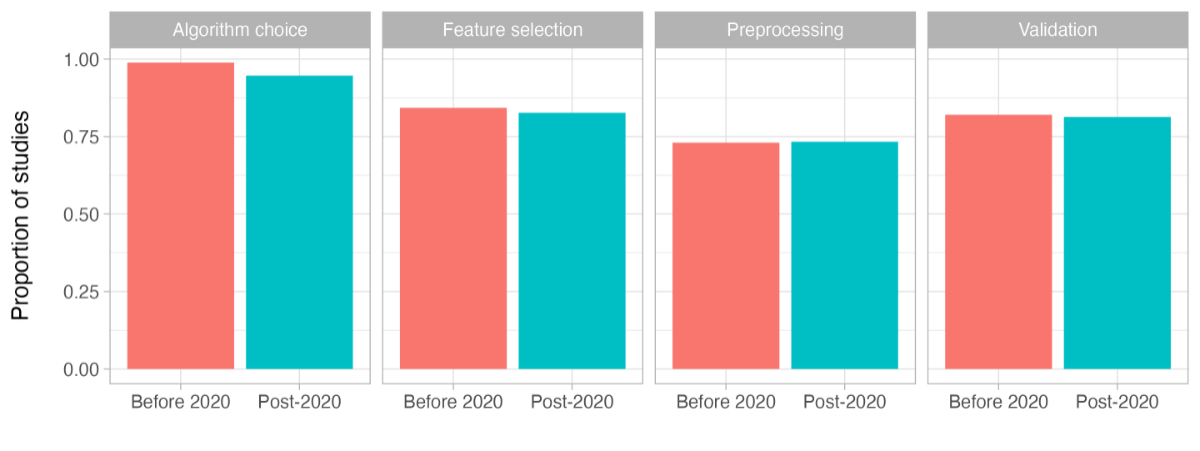
The proportion of studies that reported each of the stages of modeling that we considered, split into those published before 2020 (n=89) and those published in 2020 or later (n=75).

In summary, we found that 73.1% (120/164) of the studies described at least some of their preprocessing steps, 83.5% (137/164) described the features or feature selection process, 97% (159/164) described the algorithm or algorithms used, and 81.7% (134/164) gave some description of their model validation process. Figure 4 illustrates that there has not been much change in reporting standards since 2020, and in fact, the areas of algorithm choice and feature selection have been reported in fewer papers more recently. In the following subsections, we report on the studies that did include this information by summarizing the methodologies that were used across the literature in each stage.

#### Preprocessing

When attempting to interpret textual data using computational methods, it is typical to preprocess or *clean* textual data to prepare them for feature generation and selection. These steps tend to focus on making the text less noisy by removing data that are unlikely to be useful in the predictive task, such as stripping nonalphanumeric characters, removing stop words (common or filler words), lemmatizing the text (transforming words to their root), or tokenization (splitting sentences or documents into separate tokens delimited by spaces).

However, for data taken from social media, some preprocessing stages may be adapted to reflect the inherent meaning that, for instance, nonalphanumeric characters and stop words contribute to the text. These characteristics of text may also be expected by some sentiment analysis algorithms such as the Valence Aware Dictionary for Sentiment Reasoning [77]. Another consideration regarding internet language is the inclusion of emoji in text. Emoji often have meaning in natural language [78], and so their inclusion is likely to be relevant in textual interpretation tasks.

Two main approaches were taken by the studies that described their preprocessing stages (120/164, 73.1%) to Twitter’s native language of interaction, such as hashtags and @-mentions. One approach was to consider these part of natural language and retain this information in the tokenization stage by, for example, replacing @-mentions with an @ symbol or URLs (or web addresses) with the word “URL” [60,79,80]. Alternatively, authors chose to tokenize the text in a more traditional manner by removing all nonalphanumeric information [73,81-87]. Studies that included emoji as tokens usually did so by replacing the emoji with the word “emoji” [60,64,88] or with a unique code for each emoji [53,79,86]. Others removed emoji altogether from the text [89-91]. Variations in these preprocessing strategies indicate that there are differences in the type of information taken forward to the feature selection and modeling stages.

Some preprocessing decisions that may have affected the effectiveness of the subsequent model training processes were rarely described. For instance, it is known that personal pronouns are a useful feature in the prediction of depression [82,92]. However, personal and other pronouns may be included in stop word dictionaries (eg, the popular Natural Language Toolkit [93] stop word list) and, thus, automatically removed from the training data before any feature selection or model fitting has taken place. In addition, many of the data sets (80/119, 67.2%) used keyword or key phrase search terms to find “positive” cases for mental health disorders, but it was not made clear whether the terms used to find the data were removed from the training data set. For example, if the term “depress” used 5 times identified a user as being depressed and this term was present ≥5 times in the training data of every person who had been labeled as depressed at the modeling stage, then the model may learn that “depress” is a reliable signal for depression.

#### Features

To apply a machine learning algorithm to a data set, a series of features (also known as variables) have to be constructed. Most studies (97/164, 59.1%) used some combination of each of the feature types, as described in Table 3.

Overall, textual interpretation and textual features were the most popular. In 43.9% (72/164) of the studies, at least one form of textual interpretation was used, such as word embeddings (numeric representations of textual data), and 76.2% (125/164) used at least one type of textual structure, which tended to be either n-grams (groups of n words that appear sequentially) or term frequencies. The word embeddings used included Word2Vec [94], GloVe [95], and Bidirectional Encoder Representations from Transformers [96], with further details on the methodology used in each study available in the web-based Multimedia Appendix 1. It is worth noting that data sets built in languages other than English were often required to derive their own preprocessing and feature selection tools such as sentiment dictionaries or stop word lists because of the lack of existing software and tools readily available in their language.

**Table 3:** Overview of feature categories, the number of studies that used at least one feature from each category, and a description of the types of features they contain (N=164).

Feature type	Studies, n (%)	Description
Text interpretation	72 (43.9)	Features interpreting the meaning of the text, usually through sentiment dictionaries
Demographics	15 (9.1)	Known or algorithmically inferred demographic information
Connectivity	35 (21.3)	Features relating to the user’s social network, such as the number of followers or @-mentions
Sharing (when)	25 (15.2)	Features relating to time, such as time between tweets, tweet frequency, or times of day
Sharing (what)	25 (15.2)	Features relating to the type of content being shared, such as URLs or retweets
Textual features and structure	125 (76.2)	Structural features of the text, such as TF-IDF^a^ scores, bag of words, and language models
Keywords	39 (23.8)	Counts or distributions of keyword lists, such as medication names
Parts of speech	33 (20.1)	Labeling parts of speech or grammatical features
Images	12 (7.3)	Use of image data, such as profile pictures or shared images

^a^TF-IDF: term frequency–inverse document frequency; a statistic that reflects word importance across a group of documents.

#### Algorithms

Although different studies chose different approaches to modeling the data, most (121/164, 73.8%) used well-recognized algorithms such as support vector machines, naïve Bayes, tree-based algorithms or regression. Table 4 shows that support vector machine appeared to be the most popular algorithm. However, it was not always the primary model and often provided a baseline measure against more complex approaches such as deep learning or as part of an ensemble learning approach. Within regression, logistic regression tended to be used, which reflects the categorical nature of most of the data sets. Deep learning approaches, for instance, convolutional neural networks, have become relatively popular over time but certainly do not form the majority.

Although all but 2 studies (162/164, 98.8%) did describe the machine learning algorithm they used to produce their final model, too few studies went into sufficient detail on their hyperparameter tuning processes to include detail in this review, where hyperparameter tuning refers to the adjustments made to the values that control the model’s learning process. It was also not common for studies to justify their choice of algorithm, although the choices were appropriate.

**Table 4:** The number of studies using each type of algorithm for at least one model (N=164).

Algorithm	Studies, n (%)
Support vector machine	84 (51.2)
Tree-based	68 (41.5)
Naïve Bayes	62 (37.6)
Regression-based	52 (31.7)
Deep learning	38 (23.2)
Other^a^	55 (33.5)
Unknown	2 (1.2)

^a^Included in the “Other” category are bespoke algorithms written for this problem [68,97] as well as less popular out-of-the-box options. Examples of these are hidden Markov models [98], a Martingale framework [74], and complex decision lists [53,99].

#### Validation

Understanding the effectiveness of a machine learning model allows us to evaluate how well the algorithm might generalize to unseen data. Most often, 10- or 5-fold cross-validation was used, as well as the area under the receiver operating characteristic curve.

In total, 2 issues relevant to model validation were rarely discussed or acknowledged in the papers. Given that some of the data sets were designed to include a small number of controls in contrast to a high number of cases, some standard metrics, particularly accuracy, are likely to overrepresent how effective the algorithm is [100]. Second, the studies rarely clarified how they stratified their data for training, testing, and validation. This has implications for assessing the potential for data leakage to create bias in the model’s effectiveness and has been shown to be problematic in other applications of machine learning in digital epidemiology [101], as well as specifically creating bias in cross-validation assessment of machine learning for mental health [102].

### Ethics

The consideration of ethics approval was assessed for a subset of 61% (100/164) of the included papers as the presence of ethics board approval or discussion of ethics by authors was only included in the rubric for reporting on studies in this review for those studies found in the third search that took place in December 2021. However, this still represents all studies published in 2020 and later, from which point we had anticipated that ethical considerations should be more prevalent given the recent increase in general awareness of data ethics issues as well as previous reviews suggesting that this was an area of concern.

Overall, we found that 85% (85/100) of the papers did not discuss any ethical issues as part of their studies. In 11% (11/100) of the papers, ethical issues were discussed thoroughly or ethics approval was granted. In 4% (4/100) of the papers, a reference was made to ethics not being applicable to the study.

Although some studies (15/100, 15%) simply did not include consideration of ethics, there were examples within this of studies that directly contravened ethical guidance published by both the Association of Internet Researchers [103] and the British Psychological Society [22] regarding the use of internet data for research. This was generally by publishing tweets verbatim, sometimes along with the mental health annotation, or by publishing usernames in the paper. In addition, at least 2% (2/100) of the studies developed web applications that allowed a user timeline or tweet to be input and a prediction displayed about whether that user was experiencing the mental health disorder under consideration, although it was not always clear whether these web applications were still operational. This suggests that some research in this area was conducted by researchers with minimal training in research ethics and without suitable institutional and governance oversight.

It is true that many studies using social media data do not require ethics approval from institutional ethics boards, largely as it can be argued that they do not include “human participants” or are using data that are publicly available on the internet [104] (although whether users perceive their data as being for research use is another matter [105]). However, the nature of the research topic means that ethics are an important and complex consideration that should be at least acknowledged in the presentation of research findings [26,27].

### Replicability

Finally, we assessed the replicability of each study in terms of the quality of the details provided. For 26.8% (44/164) of the studies, we assessed that there was enough detail for the study to be replicated. A total of 31.7% (52/164) of the studies could be partly replicated, but some assumptions about methodological processes (typically the preprocessing stages) would need to be made. However, for 41.5% (68/164) of the studies, there was not enough detail provided to attempt replication of the study because of key information being missing, such as the data annotation process, the algorithm used, or the feature construction. In some cases, it was clear that publishing formats and word limits had left limited room for description, but the authors did illustrate use of external repositories on GitHub and the Open Science Framework to host more detailed methodologies or code that provided a straightforward solution to this issue.

Only 3.7% (6/164) of the studies either provided the scripts used to analyze the data or offered to make them available upon request. Alternatively, 1.2% (2/164) of the studies provided pseudocode for all stages of the model-building process as part of the article. Overall, this was an unexpectedly low rate of code sharing given the recent emphasis in both computer science and psychology on greater methodological transparency. Although some may not share code for ethical reasons, there are alternatives, such as offering to make it available upon reasonable request, which were not widely used.

## Discussion

### Principal Findings

#### Overview

This review set out to understand the current scope, direction, and trends in the prediction of mental health outcomes from Twitter data. In total, 165 papers published between 2013 and 2021 were included in the review. The number of papers published in this area has increased yearly since 2013, and 45.7% (75/164) of the included studies were published in just the 2-year span of 2020 to 2021. We sought to assess the quality of the published research from both a machine learning and mental health perspective and make recommendations that can begin to enable the creation of meaningful outputs that support aims of mental health care provision and support. In the following sections, we summarize the principal findings and contextualize them against previous work along the themes of methodological clarity and the availability of ground truth characteristics, finally looking toward developments that would support the practical applications of these algorithms in the future.

These discussions led to a series of recommendations for studies that aim to predict mental health outcomes from social media.

#### Methodological Clarity

Every study in this review used algorithmic methods for making predictions, with a wide range of novel and exciting possibilities for future development. However, the descriptions of machine learning workflows given were often poor, and a lack of clarity was a consistent theme in the results. In 11% (18/164) of the studies, there was not an adequate description of the data sets to understand the data being used, and in 26.8% (44/164) of the studies, there was no description of model preprocessing. The proportion of studies reporting these details did not increase over time.

In addition to missing out on the authors’ reasoning, poor reporting on modeling methods also reduced replicability, with only 26.8% (44/164) of the studies assessed as being replicable with the information provided. Despite recommendations to improve the description of methodologies in place since 2017 [7] and the increasing recognition of open science practices [106], we were surprised to find that only 3.7% (6/164) of the studies made their code available either open-source or upon request, when only providing code upon request would be a reasonable means of mitigating ethical concerns.

The lack of clarity often started with a poor description of the purpose of the prediction task being attempted, which has an impact on all subsequent modeling decisions and the assessment of their suitability [24,107]. It also prevents the comparison of results between papers as it is often impossible to tell whether the same or a different predictive task is being compared.

#### Availability of Ground Truth Characteristics

We found that the processes for determining what constituted a mental health disorder and, hence, the labeling of training data was validated for only 13.4% (16/119) of the primary data sets. Keyword or self-disclosure approaches were used to develop ground truth data sets for mental health outcome annotations in 67.2% (80/119) of the data sets reviewed, with keywords being highly likely to be based on the language of a particular geographical area or age group and also prone to misspellings when focusing on clinically related keywords [108]. This reasoning assumes that those who self-report mental health disorders on the web or who use certain combinations of keywords are truly experiencing the specified outcome. It also means that groups of users who were collected for “control” groups were unlikely to be true controls given the relatively high prevalence of mental health disorders in the general population [19,24]. Attempts to work with clinicians to develop a list of keywords for depression detection have also found low levels of agreement between clinicians [109], which suggests that keyword-based detection may not be a robust means of detecting genuinely depressed users. This lack of reliable, verified ground truth data about mental health outcomes is a fundamental threat to the quality of models for mental health inference. It also aligns with concerns being raised in other fields that large web-based data sets cannot replace the need for high-quality data [3,108,110].

Without validated ground truth in most data sets (103/119, 86.6%), there was no information available to characterize the data sets by key demographics such as age, gender, or cultural background. We know that expressions of mental health disorders are cultural and variable across demographic groups [111,112] and that those using social media do not represent the general population [113-115]. A lack of this information means that it is not possible to assess the impact of demographic features on model performance, and so bias may be going unnoticed. Research by Aguirre et al [116] in 2021 reinforces this after the finding that the CLPsych data set (used in 10/164, 6.1% of the studies in this review) was not representative of the population demographics of people with depression and that a classifier produced using these data set performed most poorly for people of color.

When models are created with data sets whose ground truth cannot be verified, the importance of validating the models on alternative data sets increases [21]. Shared data sets, such as the CLPsych Task 2015 [64] data set and the one by Shen et al [53], have contributed to numerous studies by providing a data set available to researchers [117] as well as providing data with which to develop novel approaches (though, as discussed by Aguirre et al [116], these data sets are unlikely to be population representative). Sharing high-quality ground truth data sets would be a beneficial next step for future developments [21]. Owing to the sensitivity of these data, we would need to think carefully about how data sharing could be managed ethically [118]. Future possibilities lie in the use of data safe havens for controlling sensitive data access and in the use of synthetic data [53], which is a developing opportunity that allows a data set with statistical properties similar to the original data to be shared without releasing the sensitive data themselves. The work of collating available data sets has been started by Harrigian et al [119] through the development of an open-source list of data sets for predicting mental health from social media, many of which are only available upon request to comply with ethical guidelines. However, data sharing is impeded by researchers sometimes not even describing the data set they are using or providing broken or out-of-date links to data repositories.

#### Toward Practical Applications

This review of the mental health outcomes covered by the 165 papers included showed that there is a considerable focus on depression and suicidality but that anxiety receives much less attention, along with serious mental health disorders such as PTSD, schizophrenia, and psychosis. Although well-being was included in the review keywords, only 0.6% (1/164) of the studies were identified that considered well-being outcomes, predicting happiness and self-esteem measured using validated scales [51]. More specific keywords related to different types of well-being may have yielded more results in this area. Although most of the focus of the data sets reviewed was on dichotomous outcomes, a future alternative is a greater focus on symptoms of disorders [120]. This has been suggested as a solution to detecting commonly comorbid illnesses that have many connected symptoms [121], an issue that has arisen in the multiclass prediction of mental health outcomes [122]. Most of the studies reviewed (147/164, 89.6%) effectively attempted to classify someone as having a mental health disorder or not, but perhaps social media may have more to offer in the tracking of web-based behaviors that are strong proxies for specific symptoms of mental health disorders. This is perhaps best illustrated by suicidality, which is a complex concept that has been effectively modeled using machine learning [123].

Another area of development that would benefit from further investigation is the use of the time-based features of Twitter data. Considering that one of the main benefits of using social media data for monitoring is the high-resolution time-series information they provide, it was surprising that only 15.2% (25/164) of the studies used any time-based features in their models, and only 0.6% (1/164) of the studies used ground truth data that were measured at more than one time point [74]. By considering Twitter data as a time series, we could approach tasks such as identifying optimal points for intervention, using methods such as change-point detection, or simply monitoring well-being over time. Having multiple instances of ground truth data for the same individual would also allow us to assess how model performance changes over time as model drift is a particularly important concern in web-based settings where language and platform features continuously adapt, potentially resulting in the degradation of a trained model over time [124]. Clinicians have so far expressed interest in using social media to measure overall symptom changes between time points rather than as a diagnostic tool [125], and so this is an area of work that requires more attention if social media data are to have a practical use in the future.

Throughout the literature, there appears to be a consensus that more meaningful and deliberate engagement with medical professionals and patients is needed to establish a direction for future research, and explorations into Patient and Public Involvement and coproduction may be effective ways of achieving these aims. Crucially, we do not yet have a broad evidence base on how patients might want to use this technology or what they would not want it to be used for as part of their care [126]. It is clear that, for the work so far to develop into a technology with real-world utility, further consultation on useful clinical applications and the ethical dilemmas presented by them will be needed [26,127,128], but this is still work to be done.

### Recommendations

On the basis of this review, we have 2 sets of recommendations. The first is for researchers in this field, building on the recommendations made by Chancellor and De Choudhury [19], which aim to increase the quality, replicability, and transparency of mental health inferences from social media (Textbox 1).

Our second set of recommendations are broader, community-level aims that focus on developing ways of working that will enable these new technologies to achieve positive outcomes (Textbox 2).

**Recommendations for researchers in this field.**

- State the prediction task being attempted. This should include whether the outcome predictions are at the user or tweet level and what the intended use of the resulting model is.
- State the mental health outcome the model will attempt to classify and how this outcome has been defined for the purpose of labeling the training data.
- State assumptions made about the mental health outcome as part of the modeling approach taken, for instance, what type of variable the outcome has been modeled as (eg, continuous or binary) or what time frame it is assumed to be detectable within.
- When creating new data sets, ensure that they are thoroughly described. We particularly recommend the use of *Data Sheets for Datasets* [129] for thorough data set reporting, which can be included as supplementary material hosted on a web-based repository that provides a permanent digital object identifier (DOI), such as the Open Science Framework or a preprint server.
- Explain the preprocessing steps in enough detail so that they can be thoroughly understood and replicated. Particular attention should be paid to whether stop word lists are used and the train, test, split stratification to ensure that they are appropriate for the prediction task being conducted.
- Where possible, conduct error analysis to explain how and why the data have been misclassified.
- Include a code and data availability statement and ensure that any crucial links to materials use a DOI.
- Include an ethics statement that describes whether the study has received ethics approval and the ethical considerations that researchers should be aware of when reading, replicating, or applying the research. The *Ethics Sheet* for this type of research developed by Mohammad [130] is particularly recommended.

**Broader, community-level recommendations.**

- Work toward an understanding of the needs of the public and patient populations who will be the subjects of the models being developed and ensure that research is advancing in line with their needs.
- Find and agree on a means by which high-quality ground truth data and trained models can be shared securely and ethically between research groups with the purpose of improving the validation of models for predicting mental health on social media.
- Maximize the benefits of what social media can add to our understanding of mental health as opposed to replacing the role of mental health professionals. In particular, the time-series nature of social media has been underexplored so far.

### Limitations

Although the best efforts were made to include all relevant papers in this review, there is always the possibility that relevant studies were missed in the systematic search process. Similarly, the search was conducted using English-language search terms, and non-English studies were not reviewed. Previous research from Kim et al [20] showed that several studies in this area have been published by teams in China, Spain, and India, which may not have been included.

This review does not go into detail about the outcomes of the studies identified, such as their results, which models appeared to be most successful, or which features were especially relevant throughout the various approaches. These are investigations that could yield useful directions for improving future models and refining the process of feature selection. Other interesting future directions would include specific reviews of the subgroups that were not included in this review, such as veterans and new mothers, and reviews that cover other social media sites, such as Reddit, that are also common venues for digital mental health sensing [19]. This could reveal whether similar concerns about research quality persist over different domains of social media mental health research.

### Conclusions

In this review, we have shown that there is a wealth of research being conducted and published on predicting mental health outcomes from Twitter, but at present, the quality of study data sets and data set descriptions is frequently poor, and most studies do not provide enough information about their analyses to understand or attempt to replicate them. For this technology to move toward being used for the benefit of the populations it is intended for, the research community needs more sources of high-quality ground truth data with clinically valid labels that can be shared ethically for benchmarking and model training. A strong partnership between researchers, clinicians, patients, and the general public is also needed to ensure that the prediction tasks being developed will be both ethically viable and clinically useful. Given the sensitivity of this research area, researchers have an ethical responsibility to ensure the transparency of machine learning methods in terms of the data and the algorithms used and precise evaluation and reporting of a model’s effectiveness.

If we can achieve our aim of using digital data to effectively model mental health, there is potential for huge advancements in our understanding, monitoring, and management of mental health conditions in the future.

## Appendix

Multimedia Appendix 1 Database search terms used in the systematic search.

Multimedia Appendix 2 List of full-text articles reviewed for inclusion, with reasons for exclusion.

Multimedia Appendix 3 Full-text data extraction details.

## Abbreviations

API: application programming interface
CLPsych: Computational Linguistics and Clinical Psychology
PTSD: posttraumatic stress disorder

## References

[1] Correia RB, Wood IB, Bollen J, Rocha LM (2020). Mining social media data for biomedical signals and health-related behavior. Annu Rev Biomed Data Sci.

[2] Loi M (2019). The digital phenotype: a philosophical and ethical exploration. Philos Technol.

[3] Ruths D, Pfeffer J (2014). Social sciences. Social media for large studies of behavior. Science.

[4] Williams ML, Burnap P, Javed A, Liu H, Ozalp S (2019). Hate in the machine: anti-black and anti-Muslimism social media posts as predictors of offline racially and religiously aggravated crime. Br J Criminol.

[5] Alizadeh M, Weber I, Cioffi-Revilla C, Fortunato S, Macy M (2019). Psychology and morality of political extremists: evidence from Twitter language analysis of alt-right and antifa. EPJ Data Sci.

[6] Prieto VM, Matos S, Álvarez M, Cacheda F, Oliveira JL (2014). Twitter: a good place to detect health conditions. PLoS One.

[7] Wongkoblap A, Vadillo MA, Curcin V (2017). Researching mental health disorders in the era of social media: systematic review. J Med Internet Res.

[8] Global Burden of Disease Study 2013 Collaborators (2015). Global, regional, and national incidence, prevalence, and years lived with disability for 301 acute and chronic diseases and injuries in 188 countries, 1990-2013: a systematic analysis for the Global Burden of Disease Study 2013. Lancet.

[9] Whiteford HA, Degenhardt L, Rehm J, Baxter AJ, Ferrari AJ, Erskine HE, Charlson FJ, Norman RE, Flaxman AD, Johns N, Burstein R, Murray CJ, Vos T (2013). Global burden of disease attributable to mental and substance use disorders: findings from the Global Burden of Disease Study 2010. Lancet.

[10] Trautmann S, Rehm J, Wittchen HU (2016). The economic costs of mental disorders: do our societies react appropriately to the burden of mental disorders?. EMBO Rep.

[11] Fekadu W, Mihiretu A, Craig TK, Fekadu A (2019). Multidimensional impact of severe mental illness on family members: systematic review. BMJ Open.

[12] (2017). Strategy for lifelong mental health research. Medical Research Council.

[13] Naslund JA, Gonsalves PP, Gruebner O, Pendse SR, Smith SL, Sharma A, Raviola G (2019). Digital innovations for global mental health: opportunities for data science, task sharing, and early intervention. Curr Treat Options Psychiatry.

[14] Russ TC, Woelbert E, Davis KA, Hafferty JD, Ibrahim Z, Inkster B, John A, Lee W, Maxwell M, McIntosh AM, Stewart R, MQ Data Science group (2019). How data science can advance mental health research. Nat Hum Behav.

[15] Torous J, Baker JT (2016). Why psychiatry needs data science and data science needs psychiatry: connecting with technology. JAMA Psychiatry.

[16] (2013). Comprehensive mental health action plan 2013-2020. World Health Organization.

[17] Solhan MB, Trull TJ, Jahng S, Wood PK (2009). Clinical assessment of affective instability: comparing EMA indices, questionnaire reports, and retrospective recall. Psychol Assess.

[18] Guntuku SC, Yaden DB, Kern ML, Ungar LH, Eichstaedt JC (2017). Detecting depression and mental illness on social media: an integrative review. Curr Opin Behav Sci.

[19] Chancellor S, de Choudhury M (2020). Methods in predictive techniques for mental health status on social media: a critical review. NPJ Digit Med.

[20] Kim J, Uddin ZA, Lee Y, Nasri F, Gill H, Subramanieapillai M, Lee R, Udovica A, Phan L, Lui L, Iacobucci M, Mansur RB, Rosenblat JD, McIntyre RS (2021). A systematic review of the validity of screening depression through Facebook, Twitter, Instagram, and Snapchat. J Affect Disord.

[21] Ernala SK, Birnbaum ML, Candan KA, Rizvi AF, Sterling WA, Kane JM, de Choudhury M (2019). Methodological gaps in predicting mental health states from social media: triangulating diagnostic signals. Proceedings of the 2019 CHI Conference on Human Factors in Computing Systems.

[22] Research Board (2021). Ethics guidelines for internet-mediated research. The British Psychological Society.

[23] Fried EI (2017). What are psychological constructs? On the nature and statistical modelling of emotions, intelligence, personality traits and mental disorders. Health Psychol Rev.

[24] Chancellor S, Baumer EP, de Choudhury M (2019). Who is the "Human" in human-centered machine learning: the case of predicting mental health from social media. Proc ACM Hum Comput Interact.

[25] Hirschfeld RM (2001). The comorbidity of major depression and anxiety disorders: recognition and management in primary care. Prim Care Companion J Clin Psychiatry.

[26] Chancellor S, Birnbaum ML, Caine ED, Silenzio VM, de Choudhury M (2019). A taxonomy of ethical tensions in inferring mental health states from social media. Proceedings of the Conference on Fairness, Accountability, and Transparency.

[27] Conway M (2014). Ethical issues in using Twitter for public health surveillance and research: developing a taxonomy of ethical concepts from the research literature. J Med Internet Res.

[28] Camerer CF, Dreber A, Holzmeister F, Ho TH, Huber J, Johannesson M, Kirchler M, Nave G, Nosek BA, Pfeiffer T, Altmejd A, Buttrick N, Chan T, Chen Y, Forsell E, Gampa A, Heikensten E, Hummer L, Imai T, Isaksson S, Manfredi D, Rose J, Wagenmakers EJ, Wu H (2018). Evaluating the replicability of social science experiments in nature and science between 2010 and 2015. Nat Hum Behav.

[29] Klein RA, Vianello M, Hasselman F, Adams BG, Adams Jr RB, Alper S, Aveyard M, Axt JR, Babalola MT, Bahník Š, Batra R, Berkics M, Bernstein MJ, Berry DR, Bialobrzeska O, Binan ED, Bocian K, Brandt MJ, Busching R, Rédei AC, Cai H, Cambier F, Cantarero K, Carmichael CL, Ceric F, Chandler J, Chang JH, Chatard A, Chen EE, Cheong W, Cicero DC, Coen S, Coleman JA, Collisson B, Conway MA, Corker KS, Curran PG, Cushman F, Dagona ZK, Dalgar I, Rosa AD, Davis WE, De Bruijn M, De Schutter L, Devos T, De Vries M, Doğulu C, Dozo N, Dukes KN, Dunham Y, Durrheim K, Ebersole CR, Edlund JE, Eller A, English AS, Finck C, Frankowska N, Freyre MÁ, Friedman M, Galliani EM, Gandi JC, Ghoshal T, Giessner SR, Gill T, Gnambs T, Gómez Á, González R, Graham J, Grahe JE, Grahek I, Green EG, Hai K, Haigh M, Haines E, Haines MP, Heffernan ME, Hicks JA, Houdek P, Huntsinger JR, Huynh HP, IJzerman H, Inbar Y, Innes-Ker ÅH, Jiménez-Leal W, John MS, Joy-Gaba JA, Kamiloğlu RG, Kappes HB, Karabati S, Karick H, Keller VN, Keller A, Kervyn N, Knežević G, Kovacs C, Krueger LE, Krueger G, Kurtz J, Lakens D, Lazarević LB, Levitan CA, Lewis, Jr. NA, Lins S, Lipsey NP, Losee JE, Maassen E, Maitner AT, Malingumu W, Mallett RK, Marotta SA, Međedović J, Mena-Pacheco F, Milfont TL, Morris WL, Murphy SC, Myachykov A, Neave N, Neijenhuijs K, Nelson AJ, Neto F, Nichols AL, Ocampo A, O’Donnell SL, Oikawa H, Oikawa M, Ong E, Orosz G, Osowiecka M, Packard G, Pérez-Sánchez R, Petrović B, Pilati R, Pinter B, Podesta L, Pogge G, Pollmann MM, Rutchick AM, Saavedra P, Saeri AK, Salomon E, Schmidt K, Schönbrodt FD, Sekerdej MB, Sirlopú D, Skorinko JL, Smith MA, Smith-Castro V, Smolders KC, Sobkow A, Sowden W, Spachtholz P, Srivastava M, Steiner TG, Stouten J, Street CN, Sundfelt OK, Szeto S, Szumowska E, Tang AC, Tanzer N, Tear MJ, Theriault J, Thomae M, Torres D, Traczyk J, Tybur JM, Ujhelyi A, Van Aert RC, Van Assen MA, Van Der Hulst M, Van Lange PA, Van ’T Veer AE, Vásquez- Echeverría A, Vaughn LA, Vázquez A, Vega LD, Verniers C, Verschoor M, Voermans IP, Vranka MA, Welch C, Wichman AL, Williams LA, Wood M, Woodzicka JA, Wronska MK, Young L, Zelenski JM, Zhijia Z, Zhejiang University of Finance and Economics (2018). Many labs 2: investigating variation in replicability across samples and settings. Adv Methods Pract Psychol Sci.

[30] Monteiro M, Keating E (2009). Managing misunderstandings: the role of language in interdisciplinary scientific collaboration. Sci Commun.

[31] James G, Witten D, Hastie T, Tibshirani R (2013). An Introduction to Statistical Learning: with Applications in R.

[32] Keyes CL (2005). Mental illness and/or mental health? Investigating axioms of the complete state model of health. J Consult Clin Psychol.

[33] Slade M (2010). Mental illness and well-being: the central importance of positive psychology and recovery approaches. BMC Health Serv Res.

[34] Kim J, Lee D, Park E (2021). Machine learning for mental health in social media: bibliometric study. J Med Internet Res.

[35] Rahman RA, Omar K, Mohd Noah SA, Mohd Danuri SN (2018). A survey on mental health detection in online social network. Int J Adv Sci Eng Inf Technol.

[36] Sundarrajan A, Aneesha M (2018). Survey on detection of metal illnesses by analysing Twitter data. Int J Eng Technol.

[37] Giuntini FT, Cazzolato MT, de Jesus Dutra dos Reis M, Campbell AT, Traina AJ, Ueyama J (2020). A review on recognizing depression in social networks: challenges and opportunities. J Ambient Intell Humaniz Comput.

[38] Edo-Osagie O, De La Iglesia B, Lake I, Edeghere O (2020). A scoping review of the use of Twitter for public health research. Comput Biol Med.

[39] Verma B, Gupta S, Goel L (2020). A survey on sentiment analysis for depression detection. Proceedings of the Advances in Automation, Signal Processing, Instrumentation, and Control.

[40] Bilal U, Khan FH (2019). An analysis of depression detection techniques from online social networks. Proceedings of the 2nd International Conference on Intelligent Technologies and Applications.

[41] Rahman RA, Omar K, Mohd Noah SA, Danuri MS, Al-Garadi MA (2020). Application of machine learning methods in mental health detection: a systematic review. IEEE Access.

[42] Le Glaz A, Haralambous Y, Kim-Dufor DH, Lenca P, Billot R, Ryan TC, Marsh J, DeVylder J, Walter M, Berrouiguet S, Lemey C (2021). Machine learning and natural language processing in mental health: systematic review. J Med Internet Res.

[43] Zunic A, Corcoran P, Spasic I (2020). Sentiment analysis in health and well-being: systematic review. JMIR Med Inform.

[44] Babu NV, Kanaga EG (2022). Sentiment analysis in social media data for depression detection using artificial intelligence: a review. SN Comput Sci.

[45] Pourmand A, Roberson J, Caggiula A, Monsalve N, Rahimi M, Torres-Llenza V (2019). Social media and suicide: a review of technology-based epidemiology and risk assessment. Telemed J E Health.

[46] Castillo-Sánchez G, Marques G, Dorronzoro E, Rivera-Romero O, Franco-Martín M, De la Torre-Díez I (2020). Suicide risk assessment using machine learning and social networks: a scoping review. J Med Syst.

[47] Beriwal M, Agrawal S (2021). Techniques for suicidal ideation prediction: a qualitative systematic review. Proceedings of the 2021 International Conference on INnovations in Intelligent SysTems and Applications.

[48] William D, Suhartono D (2021). Text-based depression detection on social media posts: a systematic literature review. Procedia Comput Sci.

[49] Skaik R, Inkpen DZ (2020). Using social media for mental health surveillance: a review. ACM Comput Surv.

[50] Ouzzani M, Hammady H, Fedorowicz Z, Elmagarmid A (2016). Rayyan-a web and mobile app for systematic reviews. Syst Rev.

[51] Mori K, Haruno M (2021). Differential ability of network and natural language information on social media to predict interpersonal and mental health traits. J Pers.

[52] Coello-Guilarte L, Ortega-Mendoza RM, Villaseñor-Pineda L, Montes-y-Gómez M (2019). Crosslingual depression detection in Twitter using bilingual word alignments. Proceedings of the Experimental IR Meets Multilinguality, Multimodality, and Interaction: 10th International Conference of the CLEF Association.

[53] Shen G, Jia J, Nie L, Feng F, Zhang C, Hu T, Chua TS, Zhu W (2017). Depression detection via harvesting social media: a multimodal dictionary learning solution. Proceedings of the 26th International Joint Conference on Artificial Intelligence.

[54] O'Dea B, Wan S, Batterham PJ, Calear AL, Paris C, Christensen H (2015). Detecting suicidality on Twitter. Internet Interv.

[55] Sawhney R, Manchanda P, Singh R, Aggarwal S (2018). A computational approach to feature extraction for identification of suicidal ideation in Tweets. Proceedings of the 2018, Student Research Workshop.

[56] AlSagri H, Ykhlef M (2020). Quantifying feature importance for detecting depression using random forest. Int J Adv Comput Sci Appl.

[57] Yazdavar AH, Mahdavinejad MS, Bajaj G, Romine W, Sheth A, Monadjemi AH, Thirunarayan K, Meddar JM, Myers A, Pathak J, Hitzler P (2020). Multimodal mental health analysis in social media. PLoS One.

[58] Birnbaum ML, Ernala SK, Rizvi AF, De Choudhury M, Kane JM (2017). A collaborative approach to identifying social media markers of schizophrenia by employing machine learning and clinical appraisals. J Med Internet Res.

[59] Coppersmith G, Leary R, Crutchley P, Fine A (2018). Natural language processing of social media as screening for suicide risk. Biomed Inform Insights.

[60] Coppersmith G, Ngo K, Leary R, Wood A (2016). Exploratory analysis of social media prior to a suicide attempt. Proceedings of the 3rd Workshop on Computational Linguistics and Clinical Psychology.

[61] He L, Luo J (2016). “What makes a pro eating disorder hashtag”: using hashtags to identify pro eating disorder tumblr posts and twitter users. Proceedings of the 2016 IEEE International Conference on Big Data.

[62] Kang K, Yoon C, Kim E (2016). Identifying depressive users in Twitter using multimodal analysis. Proceedings of the 2016 International Conference on Big Data and Smart Computing.

[63] Moulahi B, Azé J, Bringay S (2017). DARE to care: a context-aware framework to track suicidal ideation on social media. Proceedings of the 18th Web Information Systems Engineering.

[64] Resnik P, Armstrong W, Claudino L, Nguyen T (2015). The University of Maryland CLPsych 2015 shared task system. Proceedings of the 2nd Workshop on Computational Linguistics and Clinical Psychology: From Linguistic Signal to Clinical Reality.

[65] Tsugawa S, Kikuchi Y, Kishino F, Nakajima K, Itoh Y, Ohsaki H (2015). Recognizing depression from Twitter activity. Proceedings of the 33rd Annual ACM Conference on Human Factors in Computing Systems.

[66] Waheed T, Aslam M, Awais M (2019). Predicting mental-illness from Twitter activity using activity theory based context ontology. J Med Imaging Health Inform.

[67] Yin Z, Fabbri D, Rosenbloom ST, Malin B (2015). A scalable framework to detect personal health mentions on Twitter. J Med Internet Res.

[68] Zhou TH, Hu GL, Wang L (2019). Psychological disorder identifying method based on emotion perception over social networks. Int J Environ Res Public Health.

[69] Braithwaite SR, Giraud-Carrier C, West J, Barnes MD, Hanson CL (2016). Validating machine learning algorithms for Twitter data against established measures of suicidality. JMIR Ment Health.

[70] Coppersmith G, Harman C, Dredze M (2014). Measuring post traumatic stress disorder in Twitter. Proceedings of the 8th international conference on weblogs and social media.

[71] Wang T, Brede M, Ianni A, Mentzakis E (2017). Detecting and characterizing eating-disorder communities on social media. Proceedings of the Tenth ACM International Conference on Web Search and Data Mining.

[72] Burnap P, Colombo G, Amery R, Hodorog A, Scourfield J (2017). Multi-class machine classification of suicide-related communication on Twitter. Online Soc Netw Media.

[73] Jamil Z, Inkpen D, Buddhitha P, White K (2017). Monitoring tweets for depression to detect at-risk users. Proceedings of the 4th Workshop on Computational Linguistics and Clinical Psychology — From Linguistic Signal to Clinical Reality.

[74] Vioules MJ, Moulahi B, Aze J, Bringay S (2018). Detection of suicide-related posts in Twitter data streams. IBM J Res Dev.

[75] Victor DB, Kawsher J, Labib MS, Latif S (2020). Machine learning techniques for depression analysis on social media-case study on Bengali community. Proceedings of the 4th International Conference on Electronics, Communication and Aerospace Technology.

[76] Alabdulkreem E (2021). Prediction of depressed Arab women using their tweets. J Decis Syst.

[77] Hutto C, Gilbert E (2014). Vader: a parsimonious rule-based model for sentiment analysis of social media text. Proc Int AAAI Conf Web Soc Media.

[78] Guibon G, Ochs M, Bellot P (2016). From emojis to sentiment analysis. WACAI.

[79] Weerasinghe J, Morales K, Greenstadt R (2019). “Because... I was told... so much”: linguistic indicators of mental health status on Twitter. Proc Priv Enhanc Technol.

[80] Yazdavar AH, Al-Olimat HS, Ebrahimi M, Bajaj G, Banerjee T, Thirunarayan K, Pathak J, Sheth A (2017). Semi-supervised approach to monitoring clinical depressive symptoms in social media. Proc IEEE ACM Int Conf Adv Soc Netw Anal Min.

[81] Burnap P, Colombo W, Scourfield J (2015). Machine classification and analysis of suicide-related communication on Twitter. Proceedings of the 26th ACM Conference on Hypertext & Social Media.

[82] De Choudhury M, Counts S, Horvitz E (2013). Social media as a measurement tool of depression in populations. Proceedings of the 5th Annual ACM Web Science Conference.

[83] De Choudhury M, Gamon M, Counts S, Horvitz E (2021). Predicting depression via social media. Proc Int AAAI Conf Web Soc Media.

[84] Coppersmith G, Dredze M, Harman C (2014). Quantifying mental health signals in Twitter. Proceedings of the Workshop on Computational Linguistics and Clinical Psychology: From Linguistic Signal to Clinical Reality.

[85] Deshpande M, Rao V (2017). Depression detection using emotion artificial intelligence. Proceedings of the 2017 International Conference on Intelligent Sustainable Systems.

[86] Kumar A, Sharma A, Arora A (2019). Anxious depression prediction in real-time social data. Proceedings of the 2019 International Conference on Advances in Engineering Science Management & Technology.

[87] Orabi AH, Buddhitha P, Orabi MH, Inkpen D (2018). Deep learning for depression detection of Twitter users. Proceedings of the 5th Workshop on Computational Linguistics and Clinical Psychology: From Keyboard to Clinic.

[88] Resnik P, Armstrong W, Claudino L, Nguyen T, Nguyen VA, Boyd-Graber J (2015). Beyond LDA: exploring supervised topic modeling for depression-related language in Twitter. Proceedings of the 2nd Workshop on Computational Linguistics and Clinical Psychology: From Linguistic Signal to Clinical Reality.

[89] Astoveza G, Obias RJ, Palcon RJ, Rodriguez RL, Fabito BS, Octaviano MV (2019). Suicidal behavior detection on Twitter using neural network. Proceedings of the IEEE Region 10 Annual International Conference.

[90] Oyong I, Utami E, Luthfi ET (2018). Natural language processing and lexical approach for depression symptoms screening of Indonesian Twitter user. Proceedings of the 10th International Conference on Information Technology and Electrical Engineering.

[91] Chiroma F, Liu H, Cocea M (2018). Text classification for suicide related tweets. Proceedings of the 2018 International Conference on Machine Learning and Cybernetics.

[92] Reece AG, Reagan AJ, Lix KL, Dodds PS, Danforth CM, Langer EJ (2017). Forecasting the onset and course of mental illness with Twitter data. Sci Rep.

[93] Bird S, Klein E, Loper E (2019). Natural language processing with Python - analyzing text with the Natural Language Toolkit. Natural Language Toolkit.

[94] Mikolov T, Chen K, Corrado G, Dean J (2013). Efficient estimation of word representations in vector space. Proceedings of the 2013 International Conference on Learning Representations Workshop.

[95] Pennington J, Socher R, Manning C (2014). GloVe: global vectors for word representation. Proceedings of the 2014 Conference on Empirical Methods in Natural Language Processing.

[96] Devlin J, Chang MW, Lee K, Toutanova K (2019). Bert: pre-training of deep bidirectional transformers for language understanding. Proceedings of the 2019 Conference of the North American Chapter of the Association for Computational Linguistics: Human Language Technologies, Volume 1 (Long and Short Papers).

[97] Saha K, Chan L, De Barbaro K, Abowd GD, De Choudhury M (2017). Inferring mood instability on social media by leveraging ecological momentary assessments. Proc ACM Interact Mob Wearable Ubiquitous Technol.

[98] Reece AG, Danforth CM (2017). Instagram photos reveal predictive markers of depression. EPJ Data Sci.

[99] Pedersen T (2015). Screening Twitter users for depression and PTSD with lexical depression lists. Proceedings of the 2nd Workshop on Computational Linguistics and Clinical Psychology: From Linguistic Signal to Clinical Reality.

[100] Raeder T, Forman G, Chawla NV, Holmes DE, Jain LC (2012). Learning from imbalanced data: evaluation matters. Data Mining: Foundations and Intelligent Paradigms: Volume 1: Clustering, Association and Classification.

[101] Bussola N, Marcolini A, Maggio V, Jurman G, Furlanello C (2021). AI slipping on tiles: data leakage in digital pathology. Proceedings of the Pattern Recognition. ICPR International Workshops and Challenges.

[102] Tsakalidis A, Liakata M, Damoulas T, Cristea AI (2018). Can we assess mental health through social media and smart devices? Addressing bias in methodology and evaluation. Proceedings of the 2018 European Conference on Machine Learning and Knowledge Discovery in Databases.

[103] Franzke AS, Bechmann A, Zimmer M, Ess CM (2019). Internet research: ethical guidelines 3.0. Association of Internet Researchers.

[104] Townsend L, Wallace C (2016). Social media research: a guide to ethics. University of Aberdeen.

[105] Fiesler C, Proferes N (2018). “Participant” perceptions of Twitter research ethics. Soc Media Soc.

[106] Gewin V (2016). Data sharing: an open mind on open data. Nature.

[107] Cho G, Yim J, Choi Y, Ko J, Lee SH (2019). Review of machine learning algorithms for diagnosing mental illness. Psychiatry Investig.

[108] Yin Z, Sulieman LM, Malin BA (2019). A systematic literature review of machine learning in online personal health data. J Am Med Inform Assoc.

[109] Leis A, Mayer MA, Ronzano F, Torrens M, Castillo C, Furlong LI, Sanz F (2020). Clinical-based and expert selection of terms related to depression for Twitter streaming and language analysis. Stud Health Technol Inform.

[110] Schofield P (2017). Big data in mental health research - do the n s justify the means? Using large data-sets of electronic health records for mental health research. BJPsych Bull.

[111] De Choudhury M, Sharma SS, Logar T, Eekhout W, Nielsen RC (2017). Gender and cross-cultural differences in social media disclosures of mental illness. Proceedings of the 2017 ACM Conference on Computer Supported Cooperative Work and Social Computing.

[112] Loveys K, Torrez J, Fine A, Moriarty G, Coppersmith G (2018). Cross-cultural differences in language markers of depression online. Proceedings of the 5th Workshop on Computational Linguistics and Clinical Psychology: From Keyboard to Clinic.

[113] Mellon J, Prosser C (2017). Twitter and Facebook are not representative of the general population: political attitudes and demographics of British social media users. Res Politics.

[114] Sloan L (2017). Who tweets in the United Kingdom? Profiling the Twitter population using the British Social Attitudes Survey 2015. Soc Media Soc.

[115] Di Cara NH, Winstone L, Sloan L, Davis OS, Haworth CM (2022). The mental health and well-being profile of young adults using social media. NPJ Mental Health Res.

[116] Aguirre C, Harrigian K, Dredze M (2021). Gender and racial fairness in depression research using social media. Proceedings of the 16th Conference of the European Chapter of the Association for Computational Linguistics: Main Volume.

[117] Conway M, O'Connor D (2016). Social media, big data, and mental health: current advances and ethical implications. Curr Opin Psychol.

[118] Williams ML, Burnap P, Sloan L (2017). Towards an ethical framework for publishing Twitter data in social research: taking into account users' views, online context and algorithmic estimation. Sociology.

[119] Harrigian K, Aguirre C, Dredze M (2021). On the state of social media data for mental health research. Proceedings of the 7th Workshop on Computational Linguistics and Clinical Psychology: Improving Access.

[120] Aalbers G, McNally RJ, Heeren A, de Wit S, Fried EI (2019). Social media and depression symptoms: a network perspective. J Exp Psychol Gen.

[121] Borsboom D (2017). A network theory of mental disorders. World Psychiatry.

[122] Benton A, Mitchell M, Hovy D (2017). Multi-task learning for mental health using social media text. Proceedings of the 15th Conference of the European Chapter of the Association for Computational Linguistics: Volume 1, Long Papers.

[123] Ribeiro JD, Huang X, Fox KR, Walsh CG, Linthicum KP (2019). Predicting imminent suicidal thoughts and nonfatal attempts: the role of complexity. Clin Psychol Sci.

[124] Bechini A, Bondielli A, Ducange P, Marcelloni F, Renda A (2021). Addressing event-driven concept drift in twitter stream: a stance detection application. IEEE Access.

[125] Yoo DW, Birnbaum ML, Van Meter AR, Ali AF, Arenare E, Abowd GD, De Choudhury M (2020). Designing a clinician-facing tool for using insights from patients' social media activity: iterative co-design approach. JMIR Ment Health.

[126] Mikal J, Hurst S, Conway M (2016). Ethical issues in using Twitter for population-level depression monitoring: a qualitative study. BMC Med Ethics.

[127] Ford E, Curlewis K, Wongkoblap A, Curcin V (2019). Public opinions on using social media content to identify users with depression and target mental health care advertising: mixed methods survey. JMIR Ment Health.

[128] Young SD, Garett R (2018). Ethical issues in addressing social media posts about suicidal intentions during an online study among youth: case study. JMIR Ment Health.

[129] Gebru T, Morgenstern J, Vecchione B, Vaughan JW, Wallach H, Daumé III H, Crawford K (2021). Datasheets for datasets. Commun ACM.

[130] Mohammad S (2022). Ethics sheet for automatic emotion recognition and sentiment analysis. Comput Linguist.

