# Supplementary material for: Methodologies for Monitoring Mental Health on Twitter: Systematic Review
Source: J Med Internet Res. 2023 May 8;25:e42734. doi: 10.2196/42734 (PMC10203928; doi:10.2196/42734)
Supplement: Multimedia Appendix 1 [file jmir_v25i1e42734_app1.docx]

# Methodologies for Monitoring Mental Health on Twitter: Systematic Review – Systematic Search Terms

| Database/Tool | Search Query | Restrictions Used |
| --- | --- | --- |
| Scopus | TITLE-ABS-KEY( ( depress* OR bipolar* OR wellbeing OR ptsd OR "post traumatic stress disorder" OR suici* OR "mental health" OR mentalhealth OR anxi* OR "personality disorder" OR "eating disorder" OR " ED " OR "disordered eating" OR dsm* OR icd* OR ( mental AND well* ) OR ( mental AND ill* ) OR schizophren* ) AND ( twitter OR tweet* ) AND ( algorithm OR monitor* OR predict* OR detect* OR understand OR perceiv* OR "machine learning" OR "deep learning" OR "artificial intelligence" OR ai OR interpret OR character* OR classif* OR model* OR analy* OR machine OR recogni* OR sentiment ) ) | Document Type: Article, Conference Paper or Review |
| Ovid  (Medline(R) ALL,  APA PsychInfo, APA PsychArticles Full Text) | ((algorithm or predict* or detect* or understand or perceiv* or "machine learning" or "deep learning" or "artificial intelligence" or AI or interpret or character* or classif* or model* or analy* or machine or recogni* or sentiment) and (depress* or bipolar* or wellbeing or PTSD or "post traumatic stress disorder" or suici* or "mental health" or mentalhealth or anxi* or "personality disorder" or "eating disorder" or " ED " or "disordered eating" or DSM* or ICD* or (mental and well*) or (mental and ill*) or schizophren*) and (twitter or tweet*)).ab. | Restricted to keywords in abstracts, and checked: Humans, articles with abstracts, English language.  Ran ‘de-duplicate’ with preference for articles with abstracts. |
| PubMed | "algorithm*"[Title] OR "predict*"[Title] OR "detect*"[Title] OR "understand"[Title] OR "monitor*"[Title] OR "perceiv*" OR "machine learning"[Title] OR "deep learning"[Title] OR "artificial intelligence"[Title] OR "AI"[Title] OR "interpret"[Title] OR "character*"[Title] OR "classif*"[Title] OR "model*"[Title] OR "analy*"[Title] OR "machine"[Title] OR "recogni*"[Title] OR "sentiment"[Title]  AND  "twitter"[Title] OR "tweets"[Title] OR "social media"[Title] OR "social network"[Title] OR "social networking"[Title] OR "social networks"[Title]  AND  "depress*"[Title] OR "bipolar*"[Title] OR "wellbeing"[Title] OR "PTSD"[Title] OR "post traumatic stress disorder"[Title] OR "suici*"[Title] OR "mental health"[Title] OR "mentalhealth"[Title] OR "anxi*"[Title] OR "personality disorder"[Title] OR "eating disorder"[Title] OR "ED"[Title] OR "disordered eating"[Title] OR "dsm"[Title] OR "icd"[Title] OR ("mental"[Title] AND "well*"[Title]) OR ("mental"[Title] AND "ill"[Title]) OR "schizophren*"[Title] |  |
| Web of Science | #1  TS = (algorithm OR predict* OR detect* OR understand OR perceiv* OR "machine learning" OR "deep learning" OR "artificial intelligence" OR AI OR interpret OR character* OR classif* OR model* OR analy* OR machine OR recogni* OR sentiment)  AND  TS = (depress* OR bipolar* OR wellbeing OR PTSD OR "post traumatic stress disorder" OR suici* OR "mental health" OR mentalhealth OR anxi* OR "personality disorder" OR "eating disorder" OR " ED " OR "disordered eating" OR DSM* OR ICD* OR (mental AND well*) OR (mental AND ill*) OR schizophren*)  AND  (twitter OR tweet*)  # 2  TI = ((algorithm OR predict* OR detect* OR understand OR perceiv* OR "machine learning" OR "deep learning" OR "artificial intelligence" OR AI OR interpret OR character* OR classif* OR model* OR analy* OR machine OR recogni* OR sentiment)  AND  (twitter OR tweets OR "social media" OR "social network" OR "social networking" OR "social networks")  AND  (depress* OR bipolar* OR wellbeing OR PTSD OR "post traumatic stress disorder" OR suici* OR "mental health" OR mentalhealth OR anxi* OR "personality disorder" OR "eating disorder" OR " ED " OR "disordered eating" OR DSM* OR ICD* OR (mental AND well*) OR (mental AND ill*) OR schizophren*))    Searches:  #1 OR #2 | Databases= All databases  Search language=Auto |
| Google Scholar | "predict" OR "detect", "mental health" OR "mental illness" OR "depression", "Twitter" | First 10 pages of results reviewed. Only papers that appeared relevant were included in the initial list of papers. |
